# Treatment of innominate artery during thoracic endovascular aortic repair is associated with higher stroke rate

**DOI:** 10.1016/j.xjse.2025.100084

**Published:** 2025-10-10

**Authors:** Christophe Hansen-Estruch, Zdenek Novak, Emily L. Spangler, C. Mason Berry, Sabin Joseph Bozso, Adam W. Beck, Kyle W. Eudailey

**Affiliations:** a Division of Cardiovascular Surgery, University of Alabama at Birmingham, Birmingham, Ala; b Division of Vascular Surgery, University of Alabama at Birmingham, Birmingham, Ala; c University of Alabama at Birmingham, School of Medicine, Birmingham, Ala

**Keywords:** TEVAR, aortic arch, endovascular arch, arch repair

## Abstract

**Objective:**

To evaluate clinical outcomes based on method of branch vessel revascularization for thoracic endovascular aortic repair (TEVAR) with proximal landing zone (pLZ) 0 or 1 reported in the Vascular Quality Initiative (VQI).

**Methods:**

The VQI TEVAR Registry was queried for patients with a pLZ documented as 0 or 1 for any pathology during the years 2011-2024. Patients were grouped by method of branch vessel revascularization using a 3-letter code to represent the innominate, the left carotid, and left subclavian respectively. E represents endovascular treatment, O open surgical bypass, and N represents no intervention (eg, NEE represents no innominate treatment and endovascular revascularization of the left carotid and subclavian). Four groups reflecting common treatment strategies of the innominate were compared: NEE, NOO, EEE, and OOO.

**Results:**

A total of 32,490 TEVAR procedures were performed between 2011 and 2024 with 813 zone 0 pLZ and 1010 zone 1 pLZ. 54 had endovascular revascularization of all 3 branch vessels (EEE) and 207 had open revascularization of all branch vessels (OOO). Group EEE had statistically higher rates of aneurysmal disease (77.8%) than other groups (NEE 50.0%, NOO 56.7%, and OOO 62.8%). The groups requiring innominate artery intervention had a significantly higher stroke rate (EEE 20.4%, OOO 9.7%, NEE 6.9%, and NOO 3.7%). Group OOO had higher LOS.

**Conclusions:**

Patients requiring revascularization of the innominate artery during proximal TEVAR had higher stroke rates than those who did not. Whether this is related to worse underlying disease or more endovascular manipulation of the arch, and thus amenable to risk modifying techniques, needs further investigation.

**Figure:**
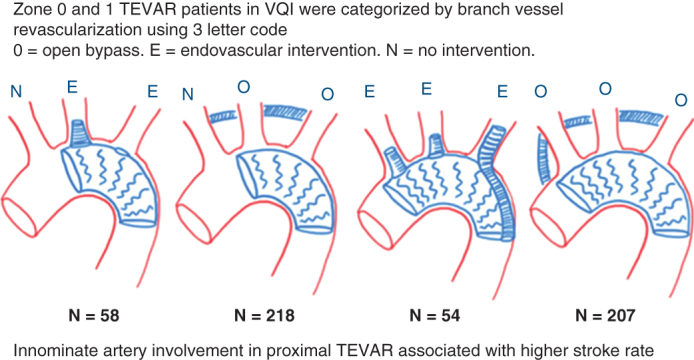
Options for revascularization after TEVAR of the aortic arch and associated stroke rates.

**Central Message**

Patients requiring revascularization of the innominate artery during proximal TEVAR had higher stroke rates than those who did not. Staged repair may confer some benefit.

**Perspective**

As ascending aorta and arch TEVAR volumes increase, understanding management of this complex population is crucial. This study addresses neurologic outcomes—a major complication limiting endovascular approaches to proximal aortic pathology—revealing that innominate artery revascularization after TEVAR is associated with higher stroke rates and establishes nomenclature for branch vessel methods.

Advances in endovascular techniques and device innovation have expanded treatment options for proximal aortic disease. Historically, repair of the proximal aorta, including ascending and arch, has been addressed with an open approach with postoperative stroke rate and operative mortality dependent on patient risk profile, case urgency, and aortic pathology. Acute type A dissection repairs can have a mortality reaching 25% and stroke rate of 13% seen in an international database with a lower mortality of 10% seen in high-volume centers with an experienced surgeon.^1^^,^^2^ Other more balanced cohorts that include 50% to 60% of patients with aneurysmal disease undergoing elective open arch procedures report early mortality ranging from 2.5% to 10% and postoperative stroke rate around 7%.^2^, ^3^, ^4^ In addition, it has been reported that up to 28% of patients with acute ascending and arch aortic pathology are deemed poor candidates for open surgery, further highlighting the risk of these procedures and the opportunity for less-invasive alternative repairs.^1^^,^^5^

During the past decade, a growing number of groups have been performing endovascular repairs of ascending and arch aortic pathology with off-label use of prior devices or, more recently, new devices specifically designed for this region of the aorta.^6^ This has given rise to several innovative endovascular and hybrid approaches to revascularize the head vessels; however, there is no consensus on the optimal method of head vessel revascularization and outcomes comparing the various approaches are lacking.^5^, ^6^, ^7^, ^8^, ^9^, ^10^, ^11^, ^12^ In-hospital mortality from these endovascular interventions is reported between 7% and 10%.^5^^,^^13^, ^14^, ^15^ Stroke rates following ascending or arch thoracic endovascular aortic repair (TEVAR) depend on the proximal landing zone with zone 0 TEVAR patients having a stroke rate of 11%.^16^

Given that endovascular repair of ascending and arch aortic pathology is still limited to several specialized centers, reporting outcomes from a large database such as the Vascular Quality Initiative (VQI) would provide a more comprehensive analysis on the effectiveness of this approach. The VQI contains multiple registries spanning 982 centers across the United States, Canada, and Singapore, including a TEVAR and complex EVAR section, making it among the largest vascular databases for these types of procedures. Thus, the primary aim of this study is to compare clinical outcomes between endovascular or hybrid methods of branch vessel revascularization following TEVAR with proximal landing zone at zone 0 or zone 1 and any distal landing zone with particular emphasis on hospital stay, mortality, and stroke rate. We anticipated that the outcomes of endovascular repair of ascending and arch aortic diseases reported from the VQI would match those reported in prior meta-analyses on the topic. We also anticipated that more extensive TEVAR or procedures requiring complex endovascular revascularization of the arch vessels would be more morbid and have higher rates of adverse neurological events but could have lower hospital lengths of stay than open repairs. Specifically, we hypothesized that involvement of the innominate artery would lead to higher stroke rate given the increased number of branch vessels treated and the greater extent of cerebral blood supply affected.

The secondary aim is to report on the current landscape of branch vessel revascularization methods recorded in the VQI, which would be illustrative for surgeons performing this procedure. We also introduce a nomenclature to describe and categorize methods of branch vessel revascularization after proximal TEVAR to create a common language system as these cases are becoming more frequent.

## Methods

### Study Population and Groups

**Figure 1:**
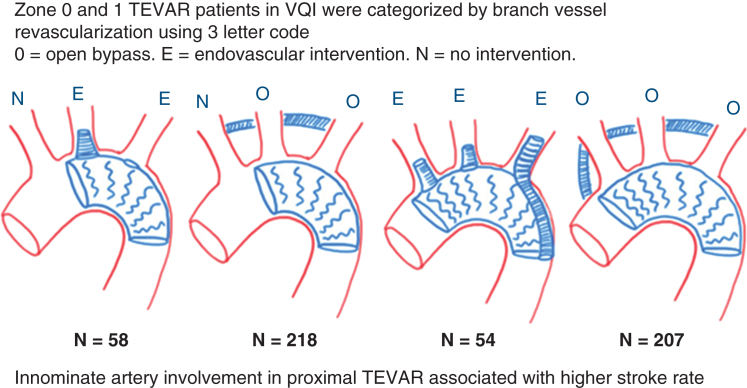
This cartoon depicts options for revascularization of the branch vessels after thoracic endovascular aortic repair (TEVAR) of the aortic arch and associated stroke rates seen in the Vascular Quality Initiative (VQI). *O*, Open bypass; *E*, endovascular; *N*, no intervention.

**Figure E1:**
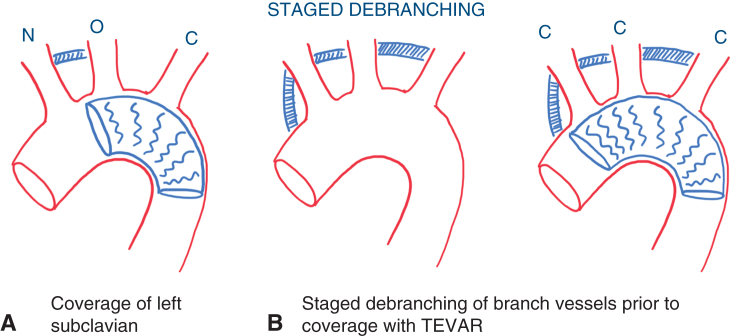
Example of covered designations in database. The diagrams represent some examples of vessel coverage which would result in a ‘C’ label in the 3-letter coding system. A, Represents intentional coverage of the left subclavian (NOC), which can be revascularized if needed at a subsequent stage. B, Represents what may be happening with the patients who are classified as ‘CCC’. It could be that the branch vessels were revascularized in a prior stage and the orifice is covered during a subsequent thoracic endovascular aortic repair (TEVAR). Because the TEVAR procedure is the only indexed procedure in the Vascular Quality Initiative (and the prior cervical debranching is not captured), the patient would be labeled ‘CCC.’ Without information that is not provided in the database, it is impossible to know how the branch vessels were addressed.

The VQI TEVAR and Complex EVAR Registry was queried for patients with a proximal landing zone documented as 0 or 1 for any pathology between 2011 and 2024. Patients with a proximal landing zone distal to zone 1 were excluded. From this, patients with distal landing zone beyond zone 0 were included. Patients were grouped based on method of branch vessel revascularization using a 3-letter code with the first letter representing the innominate, the second letter the left carotid, and the third the left subclavian. The letter E represents endovascular treatment, O represents open surgical bypass, N means the vessel was not treated (eg, NEE represents no innominate treatment and OOO represents surgical bypass to all 3 head vessels) (Figure 1). A letter C was used in the event a branch vessel was intentionally covered, such as if it were previously debranched (Figure E1). Combinations of revascularization permutations are provided in Table E1. Patients with reported coverage of the branch vessels without details of revascularization were excluded (eg, patients reported as NNN without any documentation of revascularization strategy).

We compared 4 groups that would isolate the effect of innominate artery intervention while controlling for the method of revascularization of other branch vessels. The 4 groups are: patients with complete endovascular intervention of the branch vessels (group EEE) (see Figure 1); patients without innominate intervention but endovascular approach to revascularize the left carotid and left subclavian (group NEE); patients with surgical bypass to all 3 head vessels (group OOO); and patients without intervention to the innominate and surgical bypass to the left carotid and subclavian (group NOO). Of note, the VQI does not have designations for aberrant branch vessel anatomy (eg, bovine arch), which may influence treatment categorization.

### Outcomes

Preoperative, operative, and postoperative variables were compared between these 4 groups. Missingness for variables included in this analysis was within 1%. Endoleak rate is reported as presence of any endoleak. The VQI designates prior stroke in the history section and new postintervention strokes are determined by imaging. Stroke is captured in the VQI based on cerebral distribution and type (ischemic vs hemorrhagic). We consolidated all postoperative strokes into a binary variable to indicate presence of any stroke. Length of stay is reported as median postoperative length of stay. Mortality is reported as in-hospital death.

### Statistical Analysis

Statistical analysis was performed using SPSS (IBM-SPSS Inc)) with biostatistician support within the department. Categorical data were compared using χ^2^ tests (Fisher test used when necessary). Continuous variables were compared using Student *t* tests or analysis of variance (nonparametric tests used where necessary). Logistic regression for postoperative stroke was performed using the following variables: method of branch vessel revascularization, staged repair, aneurysmal pathology, prior aneurysm repair, prior stroke, hypertension, gender, and age.

### Institutional Review Board

Due to deidentified nature of VQI data as received by us for analysis, this was considered not human subjects research by our institutional review board (#300003807) on January 3, 2020.

## Results

**Figure 2:**
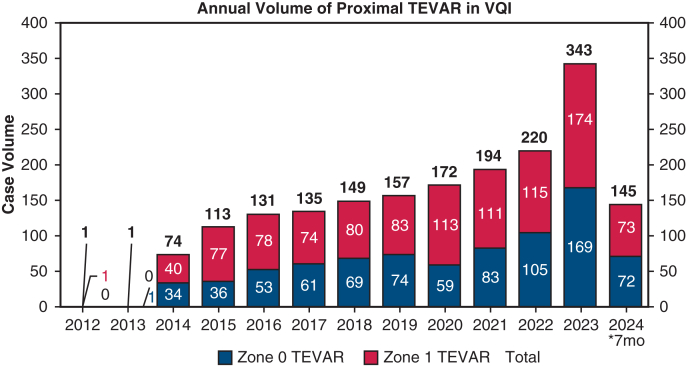
Volume of zone 0 and Zone 1 thoracic endovascular aortic repair (TEVAR) in the Vascular Quality Initiative by year. The database was queried before completion of 2024, so the final number may not represent total TEVARs performed that year.

A total of 32,490 TEVAR procedures were recorded in the VQI between 2011 and 2024 spanning 209 centers. Eight hundred thirteen (2.5%) cases had the most proximal edge of a stent land in zone 0 and 1010 (3.1%) in zone 1 for a total of 1823 cases analyzed. Volumes of TEVAR based on zone are reported per year, noting that the database was queried before the completion of year 2024 so final counts for that year are not complete (Figure 2). Fifty-six patients had isolated zone 0 TEVARs and were thus excluded because the branch vessels were not addressed. The remaining 1767 cases were categorized based on branch vessel revascularization as described above. Two hunred sixty-one patients labeled NNN (indicating no revascularization method at the time of TEVAR) were excluded from the analysis because it was impossible to assess method of branch vessel revascularization from the database.

There were 51 total permutations of repairs performed in the cohort and we selected the 4 groups with reliable data that would most logically isolate potential effects of innominate intervention on postoperative outcomes: EEE, NEE, OOO, and NOO. Fifty-four patients had endovascular revascularization of all 3 branch vessels (group EEE) and 58 had endovascular intervention on the left carotid and left subclavian (group NEE). Two hundred seven patients had open revascularization of all branch vessels (group OOO) and 218 had open intervention on the left carotid and left subclavian (group NOO). Other patients had less frequent permutations of branch vessel revascularization.

Differences in preoperative characteristics between the groups are detailed in Table 1. The average age of EEE patients was significantly older than the other groups (aged 70 years compared with 62 years [NEE], 65 years [NOO], and 64 years [OOO]; *P* = .006). In addition, the patients who underwent endovascular revascularization of the branch vessels had higher rates of prior stroke (NEE 22.4% and EEE 24.1% vs NOO 10.6% and 000 16.9%; *P* = .024). EEE patients had statistically higher rates of aneurysmal disease (77.8%) than other groups (NEE 50.0%, NOO 56.7%, and OOO 62.8%; *P* = .011).

**Table 1:** Comparison of select preoperative clinical parameters between methods of branch vessel revascularization

Preoperative variable	NEE (n = 58)	NOO (n = 218)	EEE (n = 54)	OOO (n = 207)	*P* value
Demographic characteristic					
Mean age (y)	62 ± 13	65 ± 12	70 ± 11	64 ± 12	**.006**
Male gender	36 (62.1)	146 (67.3)	34 (63.0)	148 (71.5)	.427
Caucasian	38 (65.5)	143 (65.6)	29 (53.7)	116 (56.3)	.137
Comorbidities					
ESRD	2 (3.4)	6 (2.8)	0 (0.0)	10 (4.8)	.319
Hypertension	57 (98.3)	202 (93.5)	49 (90.7)	192 (92.8)	.388
Smoking	32 (55.2)	154 (70.6)	36 (67.9)	141 (68.1)	.169
Heart failure	11 (19.0)	26 (11.9)	7 (13.0)	40 (19.3)	.159
COPD	8 (13.8)	66 (30.3)	13 (24.1)	53 (25.6)	.083
Prior CEA	1 (1.7)	6 (2.8)	3 (5.6)	4 (1.9)	.491
Diabetes	10 (17.2)	32 (14.7)	9 (16.7)	37 (17.9)	.842
Prior stroke	13 (22.4)	23 (10.6)	13 (24.1)	35 (16.9)	**.024**
Case details					
Aneurysm pathology	29 (50.0)	123 (56.7)	42 (77.8)	130 (62.8)	**.011**
Dissection	27 (46.6)	78 (35.9)	9 (16.7)	66 (31.9)	.136
Maximum aortic diameter (mm)	55	56	59	59	.170
Elective case	48 (82.8)	169 (77.5)	45 (83.3)	157 (75.8)	.522
Prior aortic aneurysm repair	30 (51.7)	78 (35.8)	23 (42.6)	73 (35.3)	.101
Prior CABG	8 (13.8)	18 (8.3)	1 (1.9)	21 (10.1)	.138
Prior PCI	15 (25.9)	26 (11.9)	9 (16.7)	19 (9.2)	**.007**

Values are presented as mean ± SD or n (%). Bolded *P*-values are considered significant. *NEE*, No intervention, endovascular, endovascular; *NOO*, surgical bypass, endovascular, endovascular; *EEE*, endovascular, endovascular, endovascular; *OOO*, surgical bypass, surgical bypass, surgical bypass; *ESRD*, end stage renal disease; *COPD*, chronic obstructive pulmonary disease; *CEA*, carotid endarterectomy; *CABG*, coronary artery bypass grafting; *PCI*, percutaneous coronary intervention.

Select intraoperative and clinical variables between the groups are presented in Table 2. Patients who underwent total endovascular repair were less likely to have a staged repair (EEE 9.3%, NEE 46.6%, NOO 51.8%, and OOO 64.3%; *P* < .001). Group EEE also had the highest rate of any endoleak at case completion (18.5% vs NEE 5.2%, NOO 9.2%, and OOO 4.8%; *P* = .003). Postoperative stroke rate was also significantly higher in the groups requiring innominate artery intervention (EEE 20.4%, OOO 9.7%, NEE 6.9%, and NOO 3.7%; *P* < .001). Group OOO had a significantly longer median length of stay (8 days vs NEE 5 days, NOO 5 days, and EEE 6 days; *P* < .001). There was no significant difference in in-hospital mortality between the groups (Table 2). A sensitivity analysis based solely on innominate intervention across groups confirmed these results as well (Table E2).

**Table 2:** Select intraoperative and postoperative clinical variables

Clinical variable	NEE (n = 58)	NOO (n = 218)	EEE (n = 54)	OOO (n = 207)	*P* value
Operative detail					
Staged repair	27 (46.6)	113 (51.8)	5 (9.3)	133 (64.3)	**<.001**
Conversion to open	0 (0.0)	1 (0.5)	2 (3.7)	6 (2.9)	.103
Any endoleak at completion	3 (5.2)	20 (9.2)	10 (18.5)	10 (4.8)	**.003**
Postoperative variable					
Reintervention on innominate	0 (0.0)	0 (0.0)	1 (1.9)	0 (0.0)	–
Reintervention on left carotid	0 (0.0)	0 (0.0)	0 (0.0)	3 (1.4)	–
Reintervention on left subclavian	0 (0.0)	10 (4.6)	0 (0.0)	3 (1.4)	–
Reintubation	5 (8.6)	16 (7.3)	3 (5.6)	26 (12.6)	.210
Stroke∗	4 (6.9)	8 (3.7)	11 (20.4)	20 (9.7)	**<.001**
Postoperative LOS (d)	5 (4, 10)	5 (3, 10)	6 (3, 12)	8 (4, 14)	**<.001**
In-hospital mortality	4 (6.9)	14 (6.4)	5 (9.3)	17 (8.2)	.852
Myocardial infarction†	1 (1.7)	2 (0.9)	0 (0.0)	2 (1.0)	.824
New dialysis need	1 (1.8)	2 (0.9)	2 (3.7)	8 (4.1)	.212
New permanent dialysis need	1 (1.8)	0 (0.0)	0 (0.0)	6 (3.0)	**.046**

Values are presented as n (%) or median (Q1, Q3). Bolded *P*-values are considered significant. *NEE*, No intervention, endovascular, endovascular; *NOO*, surgical bypass, endovascular, endovascular; *EEE*, endovascular, endovascular, endovascular; *OOO*, surgical bypass, surgical bypass, surgical bypass; *LOS*, length of stay.

∗ Any etiology and severity.

† Lab or electrocardiogram.

Given the significant difference in postoperative stroke rates between the groups, we performed a logistic regression analysis to explore the potential contributing variables (Table 3). Age, prior stroke, aneurysm pathology, method of branch vessel revascularization, and staging were included because these were significantly different across groups. We also included gender and hypertension as variables known to be associated with stroke. Results of the regression showed that method of repair and especially staging were significantly associated with strokes.

**Table 3:** Logistic regression for postoperative stroke

Variable	Odds ratio (95% CI)	*P* value
NOO vs reference∗	0.288 (0.102-0.812)	.019
Staged procedure	0.219 (0.095-0.503)	<.001

∗ Reference variable was EEE given highest observed stroke rate. Other branch groups were compared to the reference. Age, prior stroke, aneurysm pathology, method of branch vessel revascularization, and staged procedure were included in a stepwise multivariate logistic regression analysis.

Given the significance of staged repair, a subgroup analysis was performed to compare postoperative stroke rates among the branch vessel revascularization groups between those patients who underwent staged revascularization and those who underwent simultaneous repair (Table 4). Stroke rates were compared within each branch mix based on staged repair. NOO patients who underwent staged repair had significantly fewer strokes than NOO patients who had simultaneous branch vessel revascularization. This reinforces the findings seen in the logistic regression and coincides with the lowest stroke rate of all groups analyzed. Notably, none of the patients who had staged endovascular revascularization (NEE or EEE) experienced strokes, whereas simultaneous repairs in both groups did experience strokes, although the difference did not reach statistical significance.

**Table 4:** Subgroup analysis for staged procedures related to stroke

Repair method group	Simultaneous repair∗	Staged repair†	*P* value
NEE	4/31 (12.9)	0/27 (0.0)	.116
NOO	8/105 (7.6)	0/112 (0.0)	**.003**
EEE	11/49 (22.4)	0/5 (0.0)	.571
OOO	11/74 (14.9)	9/133 (6.8)	.084

Values are presented as number of patients with stroke over subgroup population (%). Bolded *P*-values are considered significant. *NEE*, No intervention, endovascular, endovascular; *NOO*, surgical bypass, endovascular, endovascular; *EEE*, endovascular, endovascular, endovascular; *OOO*, surgical bypass, surgical bypass, surgical bypass.

∗ Simultaneous repair refers to branch vessel revascularization at time of thoracic endovascular aortic repair.

† Staged repair refers to branch vessel revascularization at separate operation to thoracic endovascular aortic repair.

## Discussion

Treatment of aortic pathology, particularly of the ascending and aortic arch, has long posed a surgical challenge. Repair of this segment of the aorta is complicated by the variance in individual anatomy and the multitude of hemodynamic forces at play across the cardiac cycle. These difficulties have made endovascular intervention in the proximal aorta more complicated than the descending aorta where this approach is now routine. Nevertheless, several groups have demonstrated reproducible results using modified devices intended for the descending aorta with various methods of head vessel revascularization. In addition, endovascular devices specifically designed for use in the proximal aorta are now available. Many of these complex procedures have been performed at specialty centers with reports mainly limited to single center analysis. Meta-analyses of these single center endovascular studies have been performed and reveal stroke rates between 3.4% and 11% depending on the zone of intervention with an in hospital mortality rate of 5%.^12^^,^^16^^,^^17^ For comparison, historical stroke rates for patients who undergo open dissection repair are 13% and elective aneurysm repair are 7%.^2^ Our primary aim for this study was to use a large nationwide database to identify risk factors for stroke following branch vessel revascularization that may inform risk stratification and eventually mitigation strategies.

As a gross observation, the number of zone 0 and zone 1 TEVARs captured in the VQI is increasing yearly representing more widespread adoption of the technique (Figure 2). Again, the VQI does not capture devices that are in clinical trials and thus the majority of the initial zone 0 or zone 1 TEVARs were off-label use of abdominal devices. Notably, the Gore TBE device was approved in 2022, which may account for the uptick seen in subsequent years.^18^

As hypothesized, the patient groups that required innominate intervention (EEE, OOO) had higher stroke rates than those that did not (NOO, NEE). Prior studies have reported stroke rates for zone 1 TEVAR with any distal landing zone as 5.3%, which is comparable to the rates seen with group NEE (6.9%) and NOO (3.7%) in this study.^16^ Stroke rates for zone 0 TEVAR have been reported between 10% to 11% which is comparable to group OOO (9.7%) but lower than group EEE (20.4%). This may be expected since complete endovascular treatment would represent the highest risk patient group. Our multivariate analysis confirms that method of branch vessel revascularization was significantly associated with stroke rate. Although not controlled by randomization, these results can suggest directions for future studies for risk modification in this complex population as well as risk stratification for clinicians. Namely, if the proximal aortic repair involves revascularizing the innominate, there may be a higher stroke rate.

We recognize that extent of TEVAR itself (zone 0 vs zone 1) may account for some of this increase. To try to minimize these effects within a retrospective study, we compared stroke rates within each group based on staged repair because this was the most significant variable in our multivariate analysis. Although we were limited by small cohorts, it is quite notable that for most groups, the only strokes occurred in nonstaged patients. Without the benefit of a randomized trial, it is impossible know if this is causative, but this would suggest there is a benefit to staged repair in treating proximal aortic pathology. Other recent studies on outcomes of patients undergoing complex aortic repairs with cervical debranching report similar overall stroke rates of 4% to 11% with staged debranching observed to have an absolute reduction in stroke rate of around 10% compared with synchronous debranching (Table E3).^19^, ^20^, ^21^ Although there is no randomized trial yet, this study adds to a growing body of evidence that staged debranching, if the patient condition and anatomy allow, may represent of the best current techniques to reduce neurologic complications following complex aortic repair.

We report several methods of endovascular repair of the branch vessels in the VQI, which reveals the variability in technique. The numbers for some permutations are too low to provide meaningful comparative analysis, especially in a retrospective context. Because this area is expanding by the year, it would be beneficial to design studies to explore if there are methods of endovascular revascularization that decrease stroke rate.

Lastly, given the complexity and variance of branch vessel revascularization, we believe that the nomenclature used in this article provides a simplified and clinically useful method of describing the type of repair performed. Modifications can easily be made to incorporate method of endovascular revascularization with subscripts as desired.

### Limitations

There are natural limitations to a retrospective database study, including potential presence of confounding variables that are not captured. Also, given the complexity of these cases, there is heterogeneity in surgical methodology that cannot be accounted for in a national database. In addition, among the main limitations in the current version of the VQI is the inability to track complex patients across staged aortic procedures.

As mentioned before, the VQI captures stroke based on location and type. There is a field for clinical severity of stroke; however, there is a significant amount of missing data for this field. There can be significant heterogeneity across studies and databases regarding definition for neurologic events postoperatively that may limit cross study comparison.

Another limitation is survivorship bias inherent to the VQI TEVAR registry in that patients are only captured (and thus reported in this study) if they survived to undergo TEVAR. In other words, if patients had a multistage repair to their aorta planned but did not proceed after the first stage of debranching (eg, due to debilitating stroke or death), they would not be captured in the TEVAR registry.

Although not included in this study, comparison to patients who undergo standard open arch repair cannot be forgotten. Even with the advances in endovascular techniques and devices, open repair remains the gold standard for proximal aortic disease. There are some patient groups (eg, those with connective tissue disorders) for whom open replacement confers the benefit of durability. The expansion of endovascular options for proximal aortic disease creates an opportunity for collaboration to address these complex patients.

## Conclusions

This retrospective analysis of ascending and arch TEVAR procedures from the VQI database examined the relationship between branch vessel revascularization strategies and neurologic outcomes. Involvement of the innominate artery was associated with significantly elevated stroke rates and staged repair was associated with reduced stroke rates. These findings may inform risk stratification for patients undergoing complex aortic interventions while suggesting potential benefits of staged approaches when feasible. The establishment of a standardized nomenclature for describing branch vessel revascularization strategies, such as the 3-letter code presented in this study, may help facilitate future comparative analyses of these complex procedures as experience with proximal TEVAR continues.

### Webcast

You can watch a Webcast of this AATS meeting presentation by going to: https://www.aats.org/resources/treatment-of-innominate-artery-9767.

**Figure:**
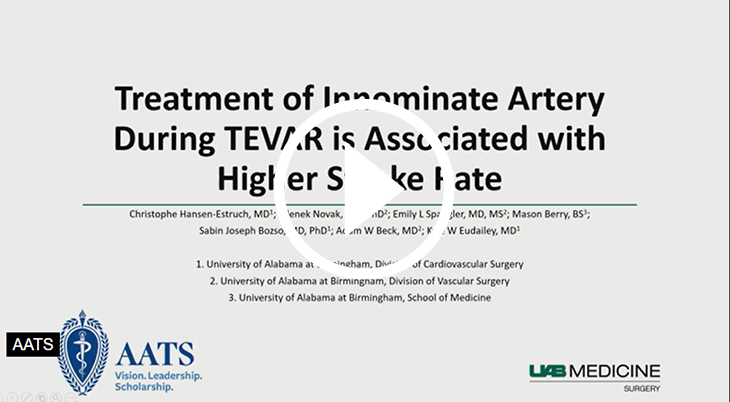

### Audio

You can listen to the discussion audio of this article by going to the supplementary material section below.

## Conflict of Interest Statement

Dr Eudailey reports speaker/advisory board roles with Terumo Aortic speaker/consultant roles with Medtronic, advisory board/consultant roles with Artivion, and speaker roles with Edwards. All other authors reported no conflicts of interest.

The *Journal* policy requires editors and reviewers to disclose conflicts of interest and to decline handling or reviewing manuscripts for which they may have a conflict of interest. The editors and reviewers of this article have no conflicts of interest.

## Figures and Tables

**Table E1:** Distribution of branch vessel revascularization in proximal aortic thoracic endovascular aortic repair (TEVAR) in the Vascular Quality Initiative (VQI)

Group	N
Incompatible with life based on available information	
NN∗	526
NC∗	57
C∗∗	24
No innominate intervention	
NEN	13
NEC	41
NEE	58
NEO	86
NON	13
NOC	104
NOE	65
NOO	218
Endo innominate intervention	
EN∗	25
EC∗	5
EEN	4
EEC	7
EEE	54
EEO	33
EON	4
EOC	18
EOE	24
EOO	85
Open innominate intervention	
ON∗	10
OC∗	2
OEE	5
OEO	2
OON	8
OOC	34
OOE	35
OOO	207
Total	1767

This table reports the distribution of branch vessel revascularization strategies following zone 0 or zone 1 TEVAR in the VQI database. Each revascularization pattern is represented by a 3-letter code corresponding to the 3 head vessels (innominate, left carotid, and left subclavian arteries, respectively). To simplify the analysis, related permutations are grouped using an ∗ to represent any letter in that position. For example, NN∗ encompasses all patterns beginning with NN (specifically NNN, NNE, NNO, and NNC) and C∗∗ encompasses all patterns beginning with C. The revascularization patterns are primarily clustered based on the approach to innominate artery management, resulting in 3 major clinical groups: no innominate intervention, endovascular innominate intervention, and open surgical innominate intervention. The groups selected for the study are in boldface type. Note that some permutations represent clinically incompatible combinations that are not compatible with patient survival, likely reflecting database limitations or data entry errors in the VQI registry.

*O*, Open bypass; *E*, endovascular; *N*, no intervention; *C*, intentionally covered vessel.

**Table E2:** Sensitivity analysis for clinical characteristics and outcomes based on innominate intervention across groups∗

Variable	N∗∗ (n = 427)	E∗∗ (n = 229)	O∗∗ (n = 291)	*P* value
Preoperative variable				
Male	278 (65.3)	149 (65.1)	198 (68.3)	.652
Caucasian	275 (64.4)	125 (54.6)	161 (55.7)	**.016**
Current smoker	111 (45.7)	58 (23.9)	217 (30.5)	.984
HTN	402 (94.6)	216 (94.3)	272 (93.5)	.817
Diabetes	72 (16.9)	29 (12.7)	45 (15.5)	.365
Prior stroke	71 (16.6)	44 (10.2)	47 (16.2)	.615
Dialysis dependent	12 (2.8)	8 (3.5)	13 (4.5)	.493
Prior CABG	44 (10.3)	16 (7.0)	32 (11.0)	.265
Prior aneurysm repair	149 (34.9)	108 (47.2)	114 (39.2)	**.009**
Elective	328 (76.8)	185 (80.8)	222 (76.3)	.411
Aneurysm	230 (54.0)	167 (72.9)	170 (58.4)	**<.001**
Postoperative variable				
MI	5 (1.2)	5 (2.2)	2 (0.7)	.309
New dialysis requirement	6 (1.4)	6 (2.7)	12 (4.3)	.068
Stroke	20 (4.7)	35 (15.3)	30 (10.3)	**<.001**
In-hospital mortality	26 (6.1)	32 (14.0)	21 (26.6)	**.002**

Values are presented as n (%). *HTN*, Hypertension; *CABG*, coronary artery bypass grafting; *MI*, myocardial infarction.

∗ This sensitivity analysis was performed across groups based only on innominate intervention. Groups that were not compatible with life based on the presented data or may have been erroneous and include: NN∗, C∗∗, NC∗, NON, NEN, NEC, NOC, EN∗, EC∗, ON∗, OC∗ were excluded from this analysis. This resulted in 947 patients analyzed. Select pre- and postoperative clinical variables are included. In brief, the groups are relatively comparable in terms of preoperative values with the exception of prior aneurysm repair and rates of aneurysmal pathology (which are reflected in the 4 studied cohorts). The differences in postoperative strokes rates are maintained and there is an increase in in-hospital mortality seen in both groups that require intervention on the innominate. Bolded p-values are considered significant.

**Table E3:** Comparison of mortality and stroke rates across studies complex aortic repairs∗

Author (y)	N	Zones involved	Pathology	Mortality info	Stroke info
Shah (2025)	29	Aortic root, ascending aorta, arch (zones 0-2)	Acute type A aortic dissection (all high-risk/inoperable, some with arch involvement)	Operative mortality 10.3% (3/29) 5-year survival 35%	Stroke 10.3% (3/29)
Khoynezhad (2016)	6	Ascending aorta (zone 0)	Type A dissection, retrograde type A dissection, intramural hematoma, penetrating ulcer, pseudoaneurysm	No early/in-hospital mortality 1 late death (16 wk, unrelated)	Stroke 17% (1/6)
Di Marco (2024)	15	Zone 0 (11/15), Zone 1 (4/15)	Aortic arch aneurysm (67%), chronic type A dissection (20%), penetrating ulcer (13%)	In-hospital mortality 6.7% (1/15) 12-mosurvival 87.5% 3 late deaths (noncardiovascular)	Perioperative stroke 13.3% (2/15) 1 additional stroke at follow-up (7.1%)
Roselli (2018)	39	Ascending aorta (zones 0A, 0B, 0C)	Acute type A dissection, chronic dissection, intramural hematoma, pseudoaneurysm, aorta-cardiac fistula	Operative mortality 13% (5/39, all in emergency type A dissection) 30 d/1 y/5 y survival: 81%/74%/64%	Stroke 10% (4/39)
Konstantinou (2019)	201	Mostly zone 2	Aneurysm, dissection, and rupture; debranching	30-d overall mortality 7.6% Staged: 4.9% Synchronous: 15%	Overall 4.3% Staged 0.7% Synchronous 13.3%
Bellamkonda (2021)	3281	Arch	Aneurysm, dissection, and rupture; debranching performed in ∼300 patients	30-day: TEVAR only 7.5% TEVAR + 1 bypass 6.8% TEVAR + 2 bypasses 22.6%	TEVAR only 3% TEVAR + 1 bypass 7.5% TEVAR + 2 bypasses 12.9%
Khoury (2025)	148	Zones 0, 1, 2	All aortic pathologies	30-day: Staged 8.0% Synchronous 2.8% (not statistically significant)	Staged 11.6%, synchronous 22.2%

∗ This table reports mortality and stroke outcomes for each of 7 studies looking at complex thoracic endovascular aortic repair in the proximal aorta, including studies that analyzed cervical debranching.

## Supplementary Data

### Audio

Audio 1 This is an audio recording of the presentation of the associated abstract and discussion. The abstract presentation ends at 7:40 and the discussion begins at 7:44.

Video 1 Video available at: https://www.jtcvs.org/article/S2950-6050(25)00043-9/fulltext.
